# The relationship between tumor markers and pulmonary embolism in lung cancer

**DOI:** 10.18632/oncotarget.17916

**Published:** 2017-05-17

**Authors:** Wei Xiong, Yunfeng Zhao, Mei Xu, Jian Guo, Bigyan Pudasaini, Xueling Wu, Jinming Liu

**Affiliations:** ^1^ Department of Cardio-Pulmonary Circulation, Shanghai Pulmonary Hospital, Tongji University School of Medicine, Shanghai, China; ^2^ Department of Respiratory Medicine, Punan Hospital, Pudong New District, Shanghai, China; ^3^ Department of Respiratory Medicine, Gongli Hospital, Pudong New District, Second Military Medical University, Shanghai, China; ^4^ Department of Pulmonary Function Test, Shanghai Pulmonary Hospital, Tongji University School of Medicine, Shanghai, China; ^5^ Department of Respiratory Medicine, Renji Hospital, Jiaotong University School of Medicine, Shanghai, China

**Keywords:** tumor markers, pulmonary embolism, lung cancer, panel of combined tumor markers, CEA

## Abstract

**Background:**

Tumor markers (TMs) and D-Dimer are both hallmarks of severity and prognosis of lung cancer. Tumor markers could be related to pulmonary embolism (PE) in lung cancer.

**Results:**

The number of abnormal tumor markers of lung cancer patients with pulmonary embolism (3.9 ± 1.1vs1.6 ± 0.6,P 0.005) was more than that in patients without pulmonary embolism. TMs panel (P trend < 0.001), CEA (R2 0.735, P0.003) and CYFRA21-1 (R2 0.718, P0.005) were positively correlated with D-Dimer in patients with pulmonary embolism. The multivariate logistic regression analysis showed that, for tumor markers, TMs panel (OR5.98, *P* < 0.001) had the strongest correlation with pulmonary embolism. The AUC (area under curve) of TMs panel and CEA were 0.82 [95%CI (0.71–0.95), *P* < 0.001] and 0.71 [95%CI (0.62–0.84), P 0.002] by ROC (receiver operating characteristic) curve analysis, respectively.

**Materials and Methods:**

Tumor markers were compared between lung cancer patients complicated with pulmonary embolism and those without pulmonary embolism Then the correlation between each tumor marker as well as panel of combined TMs and D-Dimer as well as pulmonary embolism were analyzed for patients with pulmonary embolism.

**Conclusions:**

There is a relationship between tumor markers and pulmonary embolism in patients with lung cancer. The panel of combined tumor markers is a valuable diagnostic marker for pulmonary embolism in lung cancer.

## INTRODUCTION

According to up-to-date authoritative global cancer statistics, lung cancer is the most frequently diagnosed cancer and the leading cause of cancer death among males worldwide, and among females in more developed countries [1]. In China, lung cancer is the most common incident cancer and the leading cause of cancer death [2]. The overall risk of venous thromboembolism (VTE) in cancer patients is four times as great as in the general population. Pulmonary embolism (PE) is the most serious clinical presentation of VTE. The largest absolute numbers of VTE episodes occur in patients with lung cancer. Patients receiving chemotherapy and cancer surgery have a 6-fold and 90-fold increase in risk for VTE compared with a healthy population, respectively. The 30-day all-cause mortality rates and three-month mortality rates of patients with PE is 9~11% and 8.6~17%, respectively [3]. In view of the above reasons, the diagnosis of VTE especially PE is crucial for patients with lung cancer.

It is well known that D-Dimer has been considered as a remarkable predictor associated with VTE in cancer including lung cancer [4, 5]. Interestingly, D-Dimer has been as well confirmed to be a similar predictor of lung cancer. D-dimer plasma levels that is an inexpensive, easy and noninvasive method may be useful in predicting clinical outcome, survival and treatment response of patients with lung cancer [6]. The positivity of D-dimer before and during chemotherapy was a predictor of treatment response and worse progression-free survival providing prognostic information in patients with advanced non-small cell lung cancer (NSCLC) [7]. Likewise, an elevated plasma D-dimer level could be served as an independent determinant of poor prognosis in patients with small cell lung cancer(SCLC) [8]. Meanwhile, it is common knowledge that tumor markers including CEA, CYFRA 21-1, NSE, ProGRP, and SCC especially CEA are diagnostic and prognostic hallmark of lung cancer [9–13].

Therefore, since D-Dimer and some tumor markers are both associated with the severity and prognosis of lung cancer, we postulated that tumor markers could be associated with PE in lung cancer. So far, however, there has not been any relevant literature published yet except Zhang et al. claimed that an elevated CEA level might facilitate the identification of patients at a higher risk of developing PE, whereas they also clarified simultaneously that whether or not measuring CEA levels was clinically useful for stratifying patients for PE risk needed to be made clear [14]. Moreover, the correlation between comprehensive tumor markers and PE was not discussed in the study of Zhang et al. For these reasons, this study was designed to explore the potential relationship between tumor markers and PE in lung cancer.

## RESULTS

### Demographics and characteristics of case and control groups

In total of 10618 cases of patients with lung cancer between 2015 and 2017 were recruited in three medical centers. After the inclusion and exclusion, 9527 eligible cases finally entered the study cohort. At the end of study, there were in total of 1016 cases who had a pulmonary embolism. The PE prevalence was 9.6%. Among the patients who had PE, 925 cases were symptomatic, 91 cases were asymptomatic. According to the matching, we selected 4064 patients free from PE as control group. 104 patients with PE died during hospitalization within 30 days after the diagnosis. There were another 25 deceased patients who died before the diagnostic algorithm for PE were finally diagnosed with PE by an autopsy. The detailed demographics and characteristics of case and control groups are in Table 1.

**Table 1:** Demographics and characteristics of case and control groups

Characteristic	Case(*n* = 1016)	Control (*n* = 4064)	*P* value
Age( years)	65.5 ± 18.6	63.9 ± 17.9	0.08
Female(%)	42.1%	40.9%	0.23
Male(%)	57.9%	59.1%	0.25
Smoking history(Y) (%)	70.7%	68.8%	0.07
Smoking history(N) (%)	29.3%	31.2%	0.10
SCLC(%)	12.5%	14.2%	0.18
NSCLC(%)	87.5%	85.8%	0.15
Adenocarcinoma(%)	46.7%	43.6%	0.33
Squamous(%)	28.1%	29.5%	0.19
Large cell(%)	8.6%	9.3%	0.24
Stage I(%)	18.7%	20.2%	0.26
Stage II(%)	21.6%	22.8%	0.22
Stage III(%)	28.4%	27.9%	0.17
Stage IV(%)	31.3%	29.1%	0.16
Chemotherapy(%)	76.7%	73.1%	0.38
Cancer surgery(%)	45.5%	42.9%	0.27
Anticoagulation(LCR) (%)	32.1%	34.2%	0.16
Coagulation(LCR)(%)	10.8%	11.1%	0.22
Progression(%)	78.8%	31.4%	< 0.001
Recurrence(%)	31.9%	18.2%	0.02
DVT(%)	44.9%	20.3%	0.003
D-Dimer(mg/L)	2.67 ± 1.35	0.62 ± 0.33	< 0.001

Note: (%): the proportion in case or control group; SCLC: small cell lung cancer; NSCLC: non small cell lung cancer; Stage: TNM stage; LCR: lung cancer-related; DVT: deep venous thromboembolism

### Comparison of tumor markers between case group and control group

In the comparison of each tumor marker between lung cancer patients with PE and those without PE, the results showed that, the level of CEA(18.4 ± 5.2 vs 8.1 ± 4.4, P0.006), CYFRA21-1(9.1 ± 2.7 vs 4.3 ± 2.3,P0.008) and ProGRP (92.8 ± 22.9 v s53.1 ± 18.7, P0.02) in case group were higher than those of control group. There was no difference about SCC(3.5 ± 1.3 vs 3.2 ± 1.5, P0.35) and NSE(33.5 ± 8.6 vs 31.7 ± 8.1, P 0.26) between the two groups. For the comparison about number of abnormal tumor markers in TMs panel, the results showed that, the number of abnormal tumor markers (3.9 ± 1.1 vs 1.6 ± 0.6, P 0.005) of case group was more than that of control group(Table 2).

**Table 2:** Comparison of tumor markers between case and control groups

Tumor marker	Case(*n* = 1016)	Control(*n* = 4064)	*P* value
CEA(ng/ml)	18.4 ± 5.2	8.1 ± 4.4	0.006
SCC(ng/ml)	3.5 ± 1.3	3.2 ± 1.5	0.35
CYFRA21-1(ng/ml)	9.1 ± 2.7	4.3 ± 2.3	0.008
NSE(ng/ml)	33.5 ± 8.6	31.7 ± 8.1	0.26
ProGRP(pg/ml)	92.8 ± 22.9	53.1 ± 18.7	0.02
Number of abnormal TMs(No.)	3.9 ± 1.1	1.6 ± 0.6	0.005

Note: CEA: carcinoembryonic antigen; SCC: squamous cell carcinoma antigen; CYFRA21-1:cytokeratin 19 fragment; NSE: neuron-specific enolase; ProGRP: progastrin-releasing peptide; TMs: tumor markers

### Correlation between tumor markers and D-Dimer

**Figure 1:**
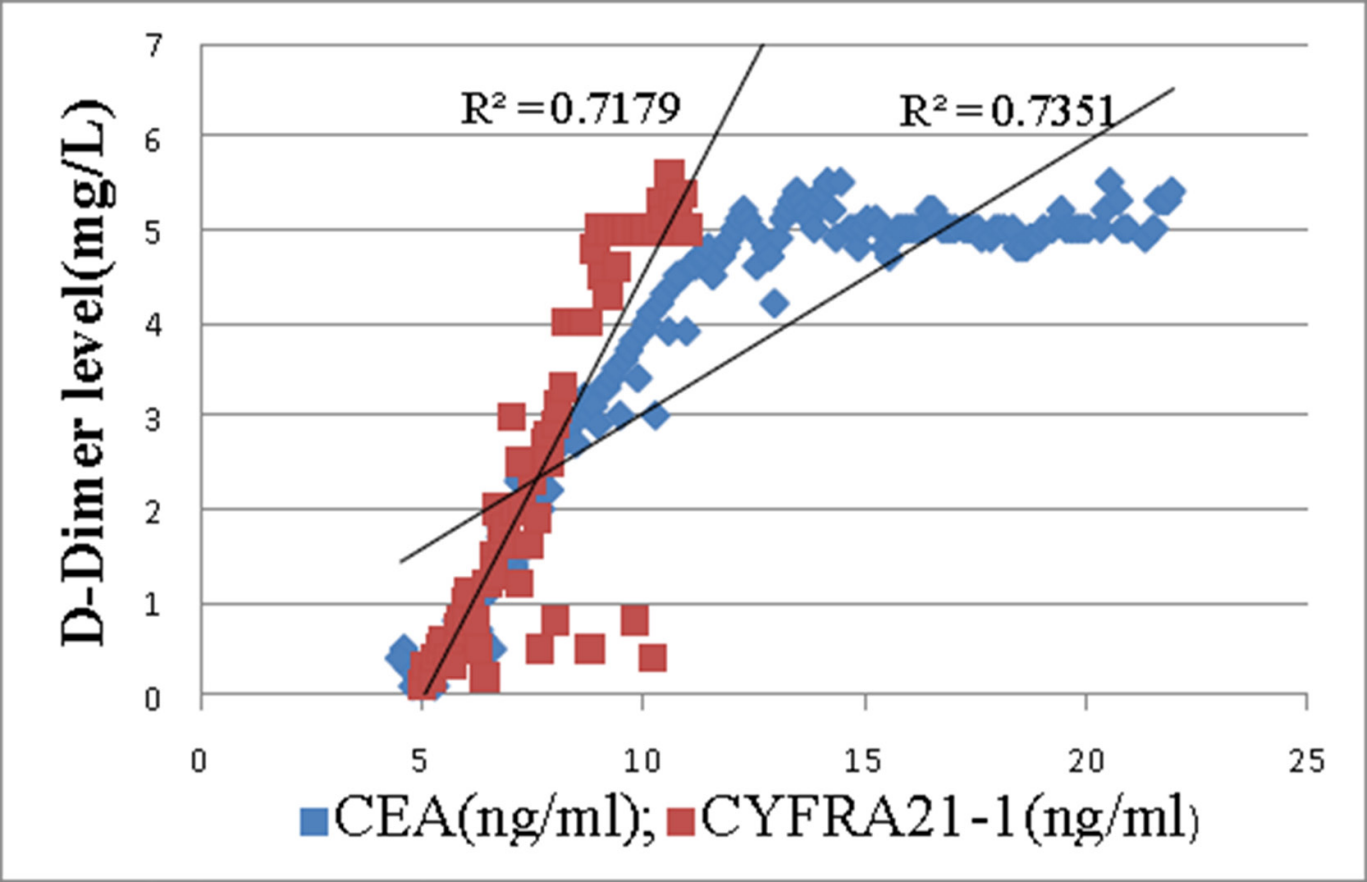
Correlation between D-Dimer and CEA(R^2^ 0.735, P0.003) as well as CYFRA21-1(R^2^ 0.718, P0.005) in case group

**Figure 2:**
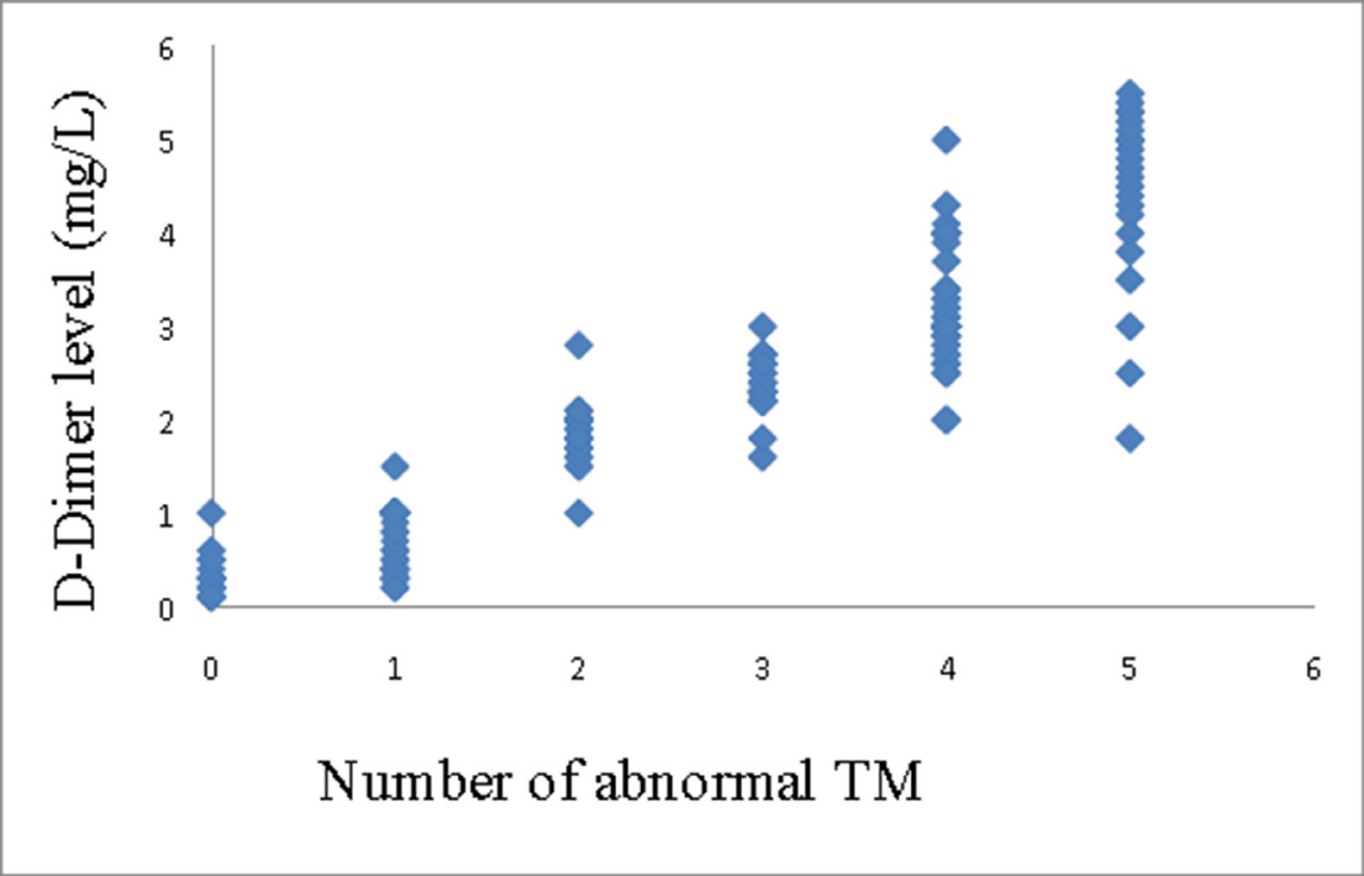
Correlation between D-Dimer and number of abnormal TMs in case group(P trend < 0.001)

The linear regression analysis between tumor markers and D-Dimer showed that, for case group, CEA(R2 0.735, P0.003) and CYFRA21-1(R^2^ 0.718, P 0.005) were correlated with D-Dimer (Figure 1), meanwhile, along with the increase of the number of abnormal TMs, the level of D-Dimer (P trend < 0.001)was increasing accordingly(Figure 2).

### Correlation between tumor markers and PE

**Figure 3:**
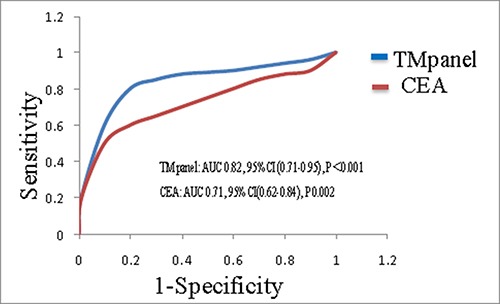
ROC curve for the diagnostic value of TMs panel and CEA in pulmonary embolism

After an univariate analysis, TMs panel (OR5.85, *P* < 0.001) and CEA (OR3.66, P 0.008) were both found to have a positive correlation with PE. The next multivariate logistic analysis demonstrated that, in tumor markers, TMs panel(OR5.98, *P* < 0.001) had the strongest correlation with PE second only to DVT(OR10.62, *P* < 0.001) and D-Dimer(OR6.16, *P* < 0.001) did, meanwhile, CEA(OR3.71, P 0.003) was the most relevant one with PE in individual TMs(Table 3). After the analysis with a ROC curve, the AUC of TMs panel was 0.82 [95%CI(0.71–0.95), *P* < 0.001], in contrast, for individual TMs, CEA which was the most correlating individual TMs demonstrated an inferior AUC of 0.71[95%CI(0.62–0.84), P 0.002], with statistical difference(P0.03)(Figure 3). We then acquired the cutoff value of how many abnormal tumor markers in TMs panel was optimal for the diagnosis of PE. The results showed that the specificity and sensitivity were 81.6% and 83.8%, respectively, when abnormal tumor markers in TMs panel were not less than 3 tumor markers.

**Table 3:** Correlation between predictors and pulmonary embolism by univariate and multivariate logistic regression analysis

	Univariate		Multivariate	
Variable	OR (95%CI)	*P* value	OR (95%CI)	*P* value
Age(yr)	1.21(0.72–2.13)	0.08	-	-
Male			1(reference)	
Female	1.52(0.88–2.26)	0.03	-	-
Smoking	history(N)		1(reference)	
Smoking history(Y)	1.16(0.63–1.92)	0.17	-	-
SCLC			1(reference)	
NSCLC	2.95(2.02–3.81)	0.01	2.88(1.97–3.78)	0.008
Adenocarcinoma	3.04(2.18–3.94)	0.003	2.93(2.19-4.14)	0.005
Squamous	1.82(0.81–3.25)	0.02	-	-
Stage I			1(reference)	
Stage II	1.23(0.66–2.19)	0.23	-	-
Stage III	2.14(1.08–3.54)	0.02	-	-
Stage IV	2.95(1.99–4.18)	0.005	2.90(1.92–4.57)	0.002
Chemotherapy	3.28(2.11–4.58)	0.007	3.32(2.24–5.17)	0.004
Cancer surgery	4.17(2.63–4.88)	< 0.001	4.33(2.36–4.92)	< 0.001
Anticoagulation(LCR)	0.83(0.26–1.54)	0.03	-	-
Coagulation(LCR)	5.35(3.97–7.96)	< 0.001	5.19(3.33–8.64)	< 0.001
Progression	2.64(1.38–4.10)	0.003	2.72(1.46–4.33)	0.007
Recurrence	2.73(1.41–4.27)	0.006	2.75(1.22–4.39)	0.001
DVT	10.09(5.28–16.34)	< 0.001	10.62(4.94–18.08)	< 0.001
D-Dimer(mg/L)	6.09(3.21–7.17)	< 0.001	6.16(3.03–8.15)	< 0.001
CEA(ng/ml)	3.66(1.87–5.43)	0.008	3.71(1.78–5.38)	0.003
SCC(ng/ml)	1.97(0.38–3.65)	0.04	-	-
CYFRA21-1(ng/ml)	2.01(1.03–3.26)	0.002	-	-
NSE(ng/ml)	1.73(0.85–2.61)	0.01	-	-
ProGRP(pg/ml)	1.89(0.73–2.94)	0.03	-	-
TMs panel(abnormal TMs)	5.85(2.78–8.97)	< 0.001	5.98(2.36–9.04)	< 0.001

Note: OR: odds ratio; SCLC: small cell lung cancer; NSCLC: non small cell lung cancer; Stage: TNM stage; LCR: lung cancer related; DVT: deep venous thromboembolism; CEA: carcinoembryonic antigen; SCC: squamous cell carcinoma antigen; CYFRA21-1:cytokeratin 19 fragment; NSE: neuron-specific enolase; ProGRP: progastrin-releasing peptide; TMs: tumor markers

## DISCUSSION

Pulmonary embolism is a common complication in lung cancer which is a leading cause of cancer-related mortality worldwide1-3. Similar as some tumor markers such as CEA, SCC, CYFRA21-1,NSE and ProGRP are, D-Dimer which is a predictor of PE is concurrently a predictor of lung cancer4-13. In that case, a hypothesis was inspired that tumor markers could be associated with PE in lung cancer. In this study, we aimed to investigate whether or not tumor markers were correlated with PE in lung cancer. To our best knowledge, this is the first study about this topic in the world so far.

In our study, we recruited the subjects from a study population in which we believed that it could represent the basic characteristics of histological classifications and PE prevalence of patients with lung cancer in China due to its large sample volume and extensive geographic distribution. Then according to the predisposing factors for PE in guidelines, we excluded confounding factors as many as possible as we could3. We also regarded recurrent hospitalizations of PE as different cases as long as they were not chronic pulmonary thromboembolism in consideration of the tumor markers of each occurrence of PE were different providing more information for analysis. Furthermore, we conducted a 1:4 approximate matching for age, sex, histology, TNM stage and oncotherapy between case and control groups to enhance the comparability and test efficiency. For tumor markers, we selected CEA, SCC, CYFRA21-1, NSE and ProGRP which had been proved to have good sensitivity and specificity in the detection of lung cancer especially in a panel of combination11. Moreover, they are most convenient, accessible and available markers in clinical setting making this study more applicable.

For the demographic and characteristic of subjects, the results showed that the age characteristics, proportion of histological classifications, and sex ratio in case and control groups, together with the PE prevalence in all patients were similar to the authoritative statistics [3, 15], meanwhile, no statistical difference were found about age, sex, smoking history, histology, TNM stage or oncotherapy between case and control groups. This means the representativeness and comparability of the subjects in this study are fairly reliable. In the comparison of individual tumor marker between lung cancer patients with a PE and those without a PE, CEA, CYFRA21-1 and ProGRP in case group were higher than those of control group. For the comparison about number of abnormal tumor markers in TMs panel, the number of abnormal TMs of case group was more than that of control group. Patients with multiple abnormal tumor markers appear to have greater chance to have a PE. For the relationship between tumor markers and D-Dimer, we found that, TMs panel, CEA and CYFRA21-1 were positively correlated with D-Dimer in case group, in contrast, there was no correlation between TMs and D-Dimer in control group. D-Dimer has been confirmed to have a positive relationship with some tumor markers and have a predictive function for the prognosis of lung cancer in a series of studies [6–8, 16–18]. Hereby we reproduced the similar results of its positive relationship with a combination of TMs, CEA and CYFRA21-1. It suggests both D-Dimer and tumor markers can be regarded as hallmarks of both cancer and pulmonary embolism. At last a multivariate logistic analysis demonstrated that, for tumor markers, TMs panel had the strongest positive correlation with PE second only to DVT and D-Dimer which were symbols of PE in patients with lung cancer. TMs panel was an independent diagnostic factor for the probability of a pulmonary embolism, meanwhile, CEA was the most relevant individual TMs that was correlated with a PE. In the analysis by a ROC curve, compared with CEA, TMs panel demonstrated superior AUC for making the diagnosis of pulmonary embolism. Moreover, we acquired a cutoff value of how many abnormal TMs in TMs panel was optimal for the diagnosis of a PE. This means as long as the number of abnormal TMs exceed certain point which was 3, patients with lung cancer will be in a potentially great danger for a complication of PE regardless of age, sex, histology, TNM stage or oncotherapy.

A profound interpretation is necessary for the results of our study. First of all, since D-Dimer that is a hallmark of VTE or PE has been confirmed to be a diagnostic and prognostic hallmark of lung cancer, theoretically, tumor markers that are diagnostic and prognostic hallmarks of lung cancer could possibly be the hallmarks of VTE or PE. This hypothesis was verified in our study. For its elucidation, we speculate that an abnormal tumor marker always suggests the poor response to oncotherapy, advancement, progression, or recurrence in lung cancer, which all further implies the high probability of blood hypercoagulable state or even tumor embolism that both can result in pulmonary embolism. In our study, the progression and recurrence rate in case group were higher than those of control group, meanwhile, the multivariate logistic regression analysis demonstrated a positive correlation between TNM stage and PE. Secondly, instead of each individual tumor marker, why was the panel of combined tumor markers most significantly associated with PE? Previous studies have proved that multiple tumor markers were more sensitive, specific and accurate predictors than individual marker was for the detection of lung cancer [11, 13, 19, 20]. In our study, the results make us infer that as long as the tumor markers are lung cancer-related, multiple markers present more precisely diagnostic power than individual marker does for the diagnosis of PE. If that is the case, the further natural question of how many abnormal markers in a panel is optimal for assisting the diagnosis of a PE comes into being, nevertheless, “the more the better” is not appropriate this time on account of requiring more abnormal markers to predict a PE will result in higher specificity and lower sensitivity, and vice versa. In the study of Molina et al, when six TMs were assessed in combination, they considered the presence of ≥ 1 abnormal TMs value as abnormal 13, whereas in our study, we discovered that ≥ 3 abnormal TMs values in CEA, SCC, CYFRA21-1, NSE, and ProGRP was the optimal cutoff value to assist the diagnosis of PE with both high sensitivity and specificity.

In the study of Zhang et al, an elevated CEA level in the tertiles was linearly associated with an increased risk of PE with borderline significance [14]. In our study, CEA was also found to have a mild diagnostic power for the probability of a PE. For individual tumor marker, CEA had the strongest relationship with PE. For its elucidation, we think that since previous studies have confirmed that the highest PE prevalence was in adenocarcinoma lung cancer [21, 22], and have as well confirmed that CEA was a relatively sensitive hallmark of adenocarcinoma lung cancer [20, 23], therefore, in other words, an elevated CEA may imply a greater probability of adenocarcinoma lung cancer, which further suggests a greater probability of pulmonary embolism. Likewise, the results of our study also showed a positive correlation between adenocarcinoma and PE. In our study, the rest individual tumor marker such as CYFRA21-1,SCC, NSE and ProGRP did not demonstrate too much close relationship with pulmonary embolism although there were some difference about CYFRA21-1 and ProGRP between case and control groups. Due to CYFRA21-1 and SCC that are considered to be markers of squamous cell lung cancer instead of adenocarcinoma lung cancer, meanwhile, NSE and ProGRP representing SCLC which accounts for only a small proportion of whole lung cancer population and has less PE prevalence than NSCLC does [24–26], the predictive power of these tumor markers could be depreciated.

For the clinical implications of this study in terms of practical measures, we believe that an elevated TMs could at least assist clinicians to take PE into account and to make an accurate diagnosis of PE in lung cancer patients. Since TMs is a routine test in clinical practice for hospitalized patients with lung cancer at least in China, it may after all possess some value for the improvement of accuracy of diagnosis of PE in lung cancer. Nevertheless, without follow-up data, of course there has not been sufficient evidence supporting prognostic evaluation value of TMs in PE related to lung cancer yet, let alone prophylaxis being recommended in lung cancer patients with elevated TMs. A follow-up study about the prognostic evaluation value of TMs and effect of prophylaxis for PE in lung cancer are warranted in the future.

### Limitations

First of all, although we excluded several factors which all might influence tumor markers or probability of PE as possible as we could, there still could be some confounding factors which had not been excluded by us. The second is that on account of there is no international standard reference or assay for tumor markers, the results of our study might not be consistent with the results of other comparable studies which adopt other criteria of reference or assay. The last one is that since our study included only Chinese patients with lung cancer, the results may not be applicable for other races.

## MATERIALS AND METHODS

### Study design

We performed a prospective, multi-centered, case-control study. Based on the inclusion criteria and exclusion criteria, we recruited the patients with lung cancer between January 2015 and January 2017 in four medical centers which treated patients with lung cancer across the whole country. Tumor markers were compared between lung cancer patients complicated with a PE and patients without a PE. Then the correlation between each tumor marker as well as panel of combined TMs and D-Dimer as well as the occurrence of PE were analyzed for patients with a PE. The participating hospitals were Shanghai Pulmonary Hospital, Shanghai Renji Hospital, Shanghai Punan Hospital, and Shanghai Gongli Hospital. This study was approved by the institutional review board of each institution. All participants signed an informed consent form.

### Patients

All patients with a confirmed histological diagnosis and staging diagnosis of lung cancer older than 18 years hospitalized between January 2015 and January 2017 were included. All patients were hospitalized either for a routine oncotherapy or a routine checkup or a suspicion for PE. All patients were admitted through emergency departments or outpatient departments. Histological types and staging (TNM) of lung cancer as well as the criteria of progression and recurrence were determined according to the international recommendations [27, 28]. The patients were excluded if they had the following cases in previous three months: pregnancy, thrombophilia, hemophilia, chronic pulmonary thromboembolism, infection, other tumors, oral contraceptive use, hormone replacement therapy, erythropoiesis-stimulating agents therapy, trauma, bone fracture, blood transfusion, immobilization longer than a week, lung cancer-irrelevant coagulation or anticoagulation or surgery. The patients with a PE during hospitalization was defined as case group. For the patient who had more than one time of hospitalization due to PE during the study period, each hospitalization with a PE would be counted as an independent case. After the performance of a 1:4 approximate matching for age, sex, histology, TNM stage and oncotherapy with patients in case group, we randomly selected certain amount of lung cancer patients without PE during the study period as control group. The detail of inclusion criteria and exclusion criteria are described in Table 4.

**Table 4:** Inclusion criteria and exclusion criteria

Inclusion Criteria	Exclusion Criteria
Patients with a confirmed histological diagnosis and staging diagnosis of lung cancer older than 18 years	Patients with one of the following cases in previous three months: 1. Chronic pulmonary thromboembolism 2. Pregnancy 3. Thrombophilia, hemophilia 4. Other tumors 5. Infection 6. Oral contraceptive use, hormone replacement therapy, erythropoiesis-stimulating agents therapy 7. Blood transfusion, trauma, bone fracture, immobilization longer than a week 8. Lung cancer-irrelevant coagulation or anticoagulation or surgery

### Assessment

**Figure 4:**
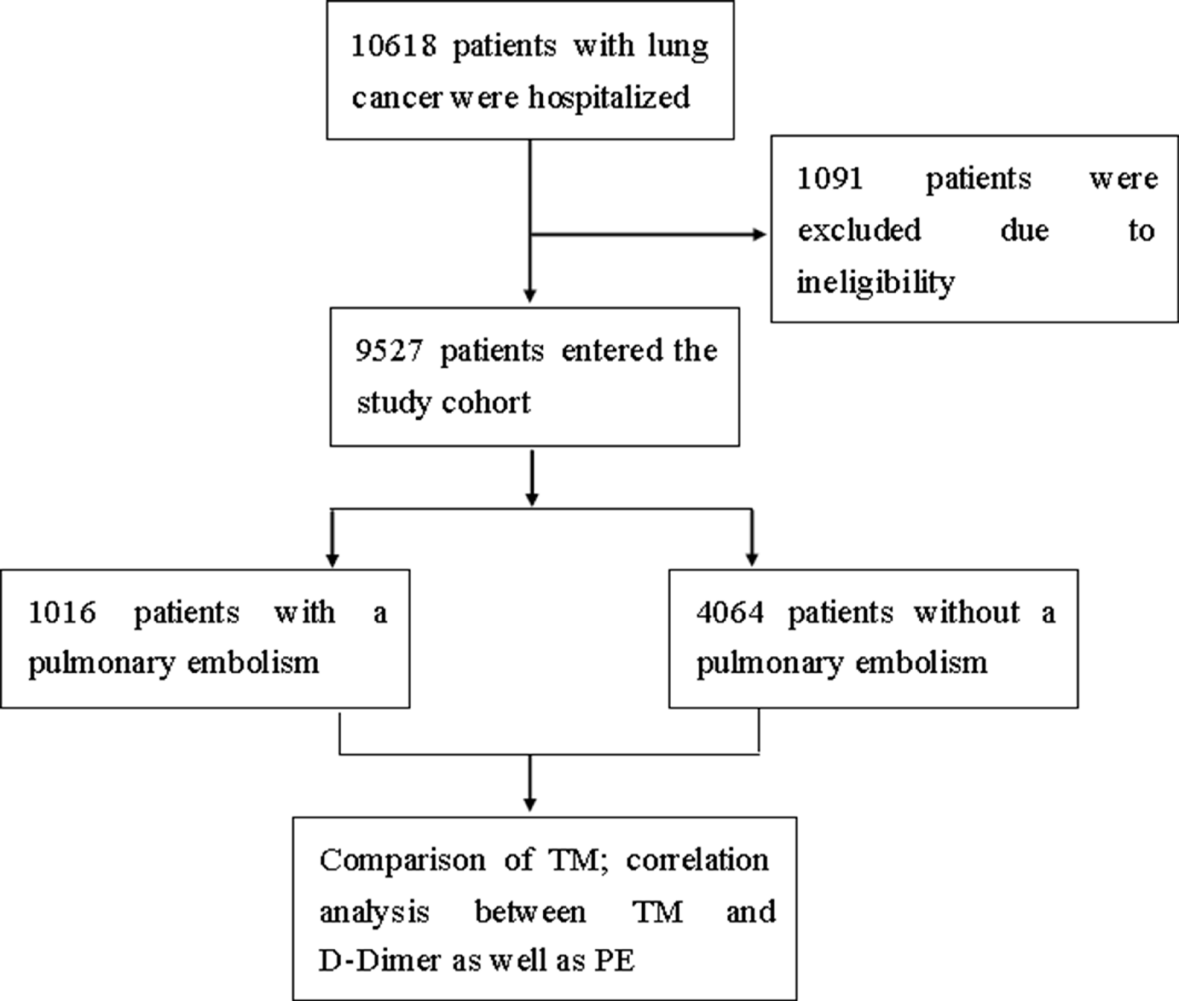
Enrollment, screening, matching and assessment

As soon as patients were admitted into hospital, the inclusion and exclusion procedure were initiated. All study assessments were completed within 24 hours after a patient was finally recruited into the study. Serum tumor markers which comprised CEA, SCC and CYFRA21-1, NSE, and ProGRP were routinely assayed at the admission for all eligible patients. The normal range of reference value of TMs were considered as the following: CEA, 0~5 ng/ml; CYFRA 21-1, 0~3.3 ng/ml; SCC, 0~2 ng/ml; NSE, 0~25 ng/ml; and ProGRP, 0~50 pg/ml according to the previously published literatures [29, 30]. Any individual TMs value above the upper limit of normal range was considered abnormal. The definition of the panel of combined tumor markers was the panel of all five tumor markers, meaning there could be more than one abnormal tumor marker value for a patient. D-Dimer was also assayed at the admission. For lung cancer patients with a suspected PE, computed tomographic pulmonary angiography was performed, or a ventilation–perfusion lung scanning in the case of patients with allergy to contrast material, or a pulmonary angiography of right heart catheterization in the case of diagnosis was difficult to be confirmed through the abovementioned methods. The criterion for the diagnosis of pulmonary embolism was an intraluminal filling defect on computed tomography or a perfusion defect of at least 75% of a segment with corresponding normal ventilation [31, 32]. In the case that a suspected patient died before the completion of diagnostic algorithm for PE, an autopsy was requested. All tumor markers and D-Dimer were measured with an electro-chemiluminiscent assay and an enzyme-linked immunosorbent assay, respectively (ROCHE Diagnostics, Basel, Switzerland). The flowchart of enrollment, screening, matching and assessment of this study are in Figure 4.

### Statistics

According to whether or not it conforms to a normal distribution, measurement data was presented as mean ± standard deviation or median with interquartile range. Categorical data was presented as frequencies and percentages. Comparison of continuous variables between two groups was conducted with a *t*-test. Comparison of rate between two groups was conducted with a Chi-square test. We performed an univariate and a multivariate logistic regression analyses to calculate odds ratio (OR) and 95% confidence intervals (CI) for each parameter as a variable. The diagnostic value was assessed by a ROC curve. Statistical significance was accepted at *P* < 0.05. All statistical analyses were conducted with a SPSS 22 software (SPSS Inc., Chicago, IL, USA).

## CONCLUSIONS

There is a relationship between tumor markers and pulmonary embolism in patients with lung cancer. The panel of combined tumor markers is a valuable diagnostic marker for pulmonary embolism in lung cancer. The diagnosis of pulmonary embolism should be considered if there are not less than three abnormal tumor markers in a panel of CEA, SCC, CYFRA21-1, NSE, and ProGRP in patients with lung cancer. We hope that this finding could shed some new light on clinical implications for the diagnosis of pulmonary embolism in lung cancer.
